# Bladder paraganglioma treated with open partial cystectomy: a case report

**DOI:** 10.1186/s13256-022-03715-x

**Published:** 2022-12-25

**Authors:** Tugay Aksakallı, Bakytbek Kozubaev, Turgut Yapanoğlu, Adem Utlu, Fatih Alper, Arzu Bilen, Numan Bulut

**Affiliations:** 1 Department of Urology, Erzurum Regional Training and Research Hospital, University of Health Sciences, Istanbul, Turkey; 2 Department of Urology, Ataturk University Medical Faculty, Erzurum, Turkey; 3 Department of Radıology, Ataturk University Medical Faculty, Erzurum, Turkey; 4 Department of Endocrınology, Ataturk University Medical Faculty, Erzurum, Turkey; 5 Department of Pathology, Ataturk University Medical Faculty, Erzurum, Turkey

**Keywords:** Paraganglioma, Partial cystectomy, Pheochromocytoma

## Abstract

**Background:**

Bladder paraganglioma is a neuroendocrine tumor that accounts for less than 0.1% of all bladder tumors. Symptoms caused by catecholamine release such as hypertension, palpitation, syncope, and macroscopic hematuria are the most common findings. Treatment modalities include transurethral resection, and partial or total cystectomy.

**Case presentation:**

A 38-year-old Turkish female patient was examined for hematuria that had been persisting for 6 months. Among the clinical findings, only hematuria was present. Absence of adrenergic symptoms such as hypertension, palpitations, and syncope at the first presentation made it difficult to consider bladder paraganglioma in the differential diagnosis. Therefore, cystoscopy and transurethral resection were performed with the thought of urothelial cancer. Findings such as hypertension and bradycardia that developed during diagnostic transurethral resection suggested that it might be bladder paraganglioma. After the radiological evaluation and endocrinological preparation, the patient underwent partial cystectomy.

**Conclusion:**

The rarity of cases having been reported in the literature leads to uncertainties in the management of bladder paraganglioma. Adrenergic symptoms developing during transurethral resection should suggest paraganglioma in the differential diagnosis. A multidisciplinary approach and medical treatment are mandatory to prevent life-threatening complications such as hypertensive crisis, vascular collapse, and multiple-organ system failure. We aimed to report the clinical presentation that includes only macroscopic hematuria mimicking urothelial cancer and to emphasize the multidisciplinary approach in the treatment.

## Background

Bladder paraganglioma is a very rare malignant tumor originating from chromaffin cells and accounting for 0.06% of all bladder tumors [1]. As seen in urothelial carcinoma, painless macroscopic hematuria may be the only symptom. In addition to macroscopic hematuria, findings such as hypertension, syncope, and palpitations during voiding can be seen due to the release of catecholamine [2].

As a diagnosis and treatment method, the transurethral approach is applied as the first step. The tumoral lesion seen during cystoscopy can be excised by transurethral resection or holmium laser ablation. These methods can also be used in combination with the laparoscopic approach.

Radical cystectomy is the standard treatment for bladder malignancies, especially muscle-invasive bladder tumors. The morbidities brought by the operation in patients with comorbidities have led to the search for alternatives to radical cystectomy. In selected cases, partial cystectomy can be performed by open, laparoscopic, and robotic methods with acceptable oncological results.

## Case presentation

A 38-year-old Turkish female patient was hospitalized for macroscopic hematuria persisting for 6 months. In the patient’s medical, family, and psychosocial history, it was found that she had undergone an operation due to multinodular goiter 7 years ago, her parents were healthy and had no history of chronic disease, and she was a housewife with two children. There were no symptoms related to the adrenergic system such as hypertension, palpitation, syncope, or flushing. Urogenital system examination findings were normal.

**Fig. 1:**
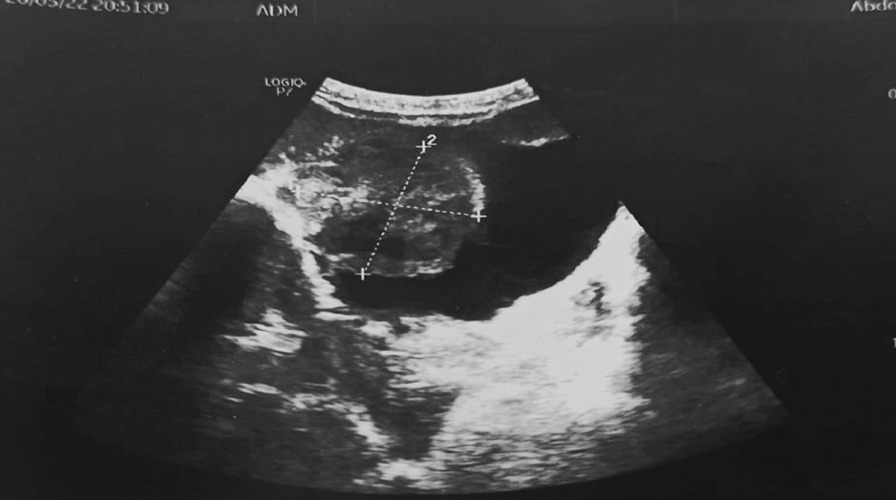
Gray-scale ultrasound image of the urinary bladder shows a calcified heterogeneous 62 × 54 mm mass along the right lateral wall of the bladder

In the laboratory tests, the fasting blood sugar was 107 mg/dl, and hemoglobin was slightly elevated at 14.2 g/dl. Microscopic hematuria was observed in the urine analysis. Urinary system ultrasonography showed that both kidney sizes and calyceal system were normal, and a solitary tumoral formation of approximately 5 × 6 cm in size, starting from the bladder dome and extending to the right lateral wall was detected (Fig. 1).

After the detection of the mass in the bladder, cystoscopy was planned under general anesthesia. Cystoscopy confirmed a 5 × 6 cm solitary tumoral lesion starting from the bladder dome and extending to the right lateral wall. A sample was obtained with a transurethral resectoscope for pathological diagnosis. During the resection of the lesion, blood pressure increased to 206/124 mmHg and heart rate increased to 189/minute. After the procedure, the patient’s blood pressure and heart rate were monitored carefully. Her vital signs were stable in the follow-ups. The cardiology clinic suggested an endocrinology consultation, stating that her vital signs were stable and that she might have a paraganglioma.

**Fig. 2:**
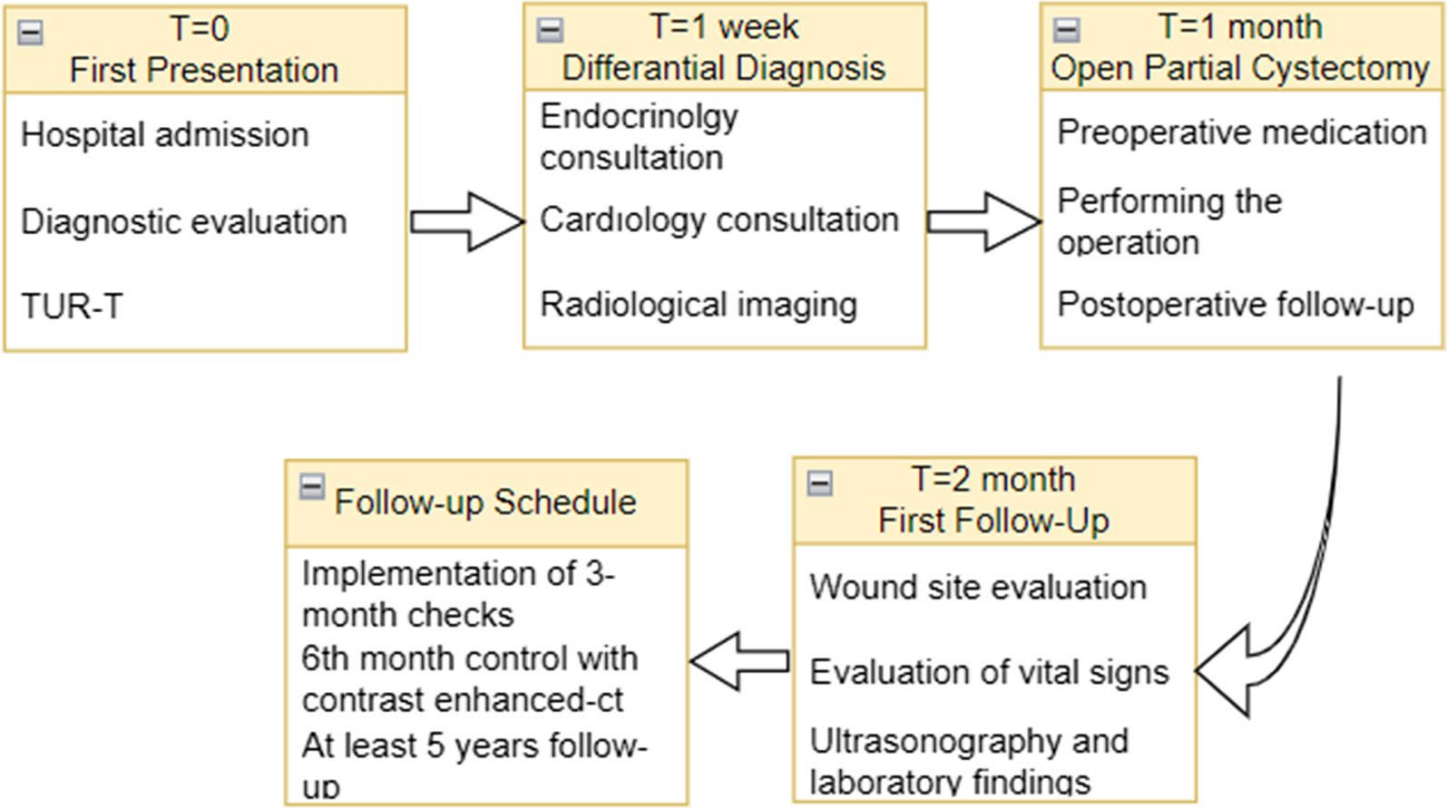
Summary of case clinic management

Subsequently, an endocrinological evaluation was performed for extra-adrenal pheochromocytoma (Fig. 2). A 24-hour urine analysis was performed at the endocrinology consultation. It was determined that metanephrine and normetanephrine levels were elevated to 974 mcg and 1857 mcg, respectively. Hence, the observed mass was in favor of extra-adrenal pheochromocytoma. In the staging tomography performed by the urology clinic, an open operation was decided upon. Therefore, to prevent the recurrence of hypertensive crisis and the development of vascular collapse, it was planned to perform the operation after 3 weeks of treatment with the alpha-blocker doxazosin. The doxazosin starting dose was 2 mg within the 3 weeks, which was increased to 16 mg.

**Fig. 3:**
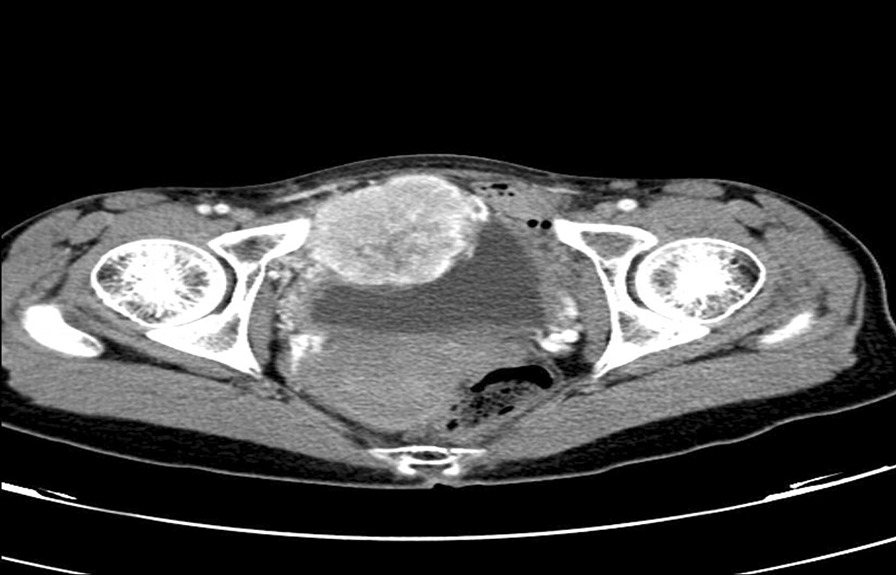
Image of a 66 × 55 mm heterogeneous, contrast-enhancing, and lobulated mass lesion starting from the anterior wall of the bladder and extending to the right lateral wall in abdominal tomography

The resection pathology was reported as cystitis. Mass boundaries and neighborhoods were evaluated with contrast-enhanced computerized tomography (CT). Contrast-enhanced tomography sections revealed a hyperdense lobulated mass lesion with a heterogeneous internal structure starting from the anterior wall of the bladder and extending to the right lateral wall, measuring approximately 66 × 55 mm (Fig. 3). There was no evidence of lymphadenopathy.

**Fig. 4:**
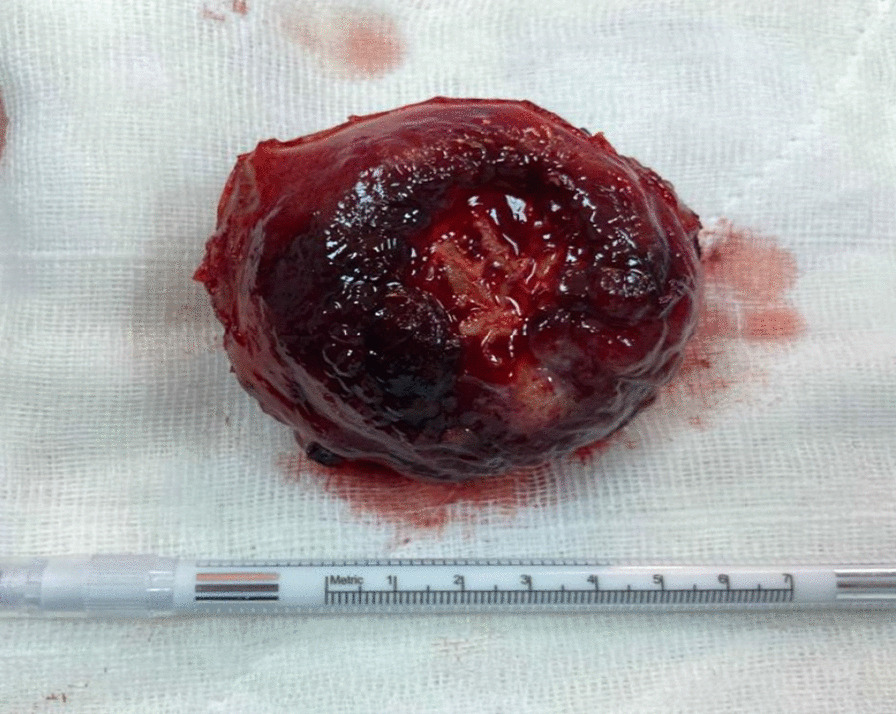
Macroscopic appearance of bladder paraganglioma: a solitary, red, lobulated tumoral formation of 6 × 5 cm diameter

After endocrinological evaluation and alpha-blocker treatment, we decided to perform an open partial cystectomy. During the dissection of the mass, blood pressure increased to 216/117 mmHg and heart rate to 223/minute. The mass was excised from the bladder to a tumor-free area of approximately 1 cm (Fig. 4). When the excision was completed, blood pressure values decreased to 56/24 mmHg and heart rate to 24/minute. Vital signs stabilized after intravenous hydration and administration of sympathomimetic agents. In the postoperative period, blood pressure values continued to be normal and no surgery-related complications developed. The transurethral catheter was removed on the tenth postoperative day.

**Fig. 5:**
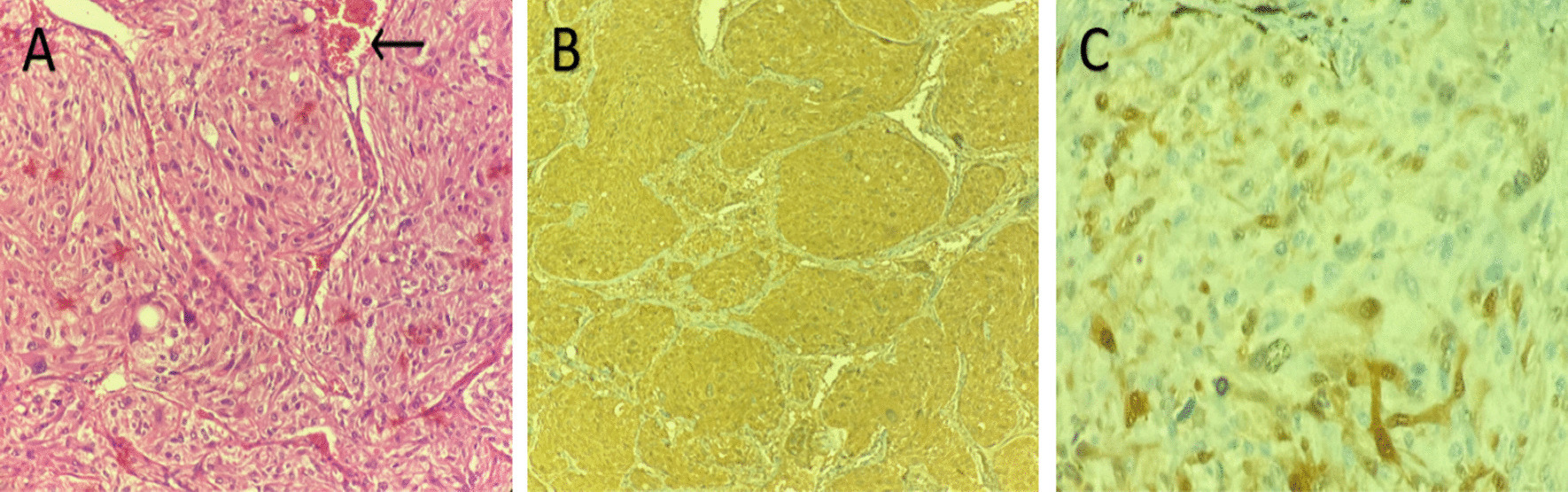
Hematoxylin and Eosin staining of bladder paraganglioma tumor (**A**) showing characteristic nests of cells (zellballen) with eosinophilic granular cytoplasm, surrounded by a prominent vascular network. Chromogranin staining (**B**) was diffusely positive, while S100 staining (**C**) was positive in sustentacular cells

The pathological evaluation of the tumor confirmed the diagnosis of vesical paraganglioma. Chromogranin, synaptophysin, S100, and gata-3 were positive on immunohistochemical staining (Fig. 5). There was no evidence of local tumor invasion.

The first control after the operation was carried out in the first month. On physical examination, the wound site was evaluated and wound healing was considered normal. Her blood pressure was 117/46 mmHg, complete blood count and biochemical values were normal, and there was no pathological finding on the urinary ultrasonography. No additional medication or recommendation was specified in the endocrinological consultation.

The follow-up of the patient continues at 3-month intervals. It is planned to evaluate the possibility of recurrence and metastasis with contrast-enhanced CT and cystoscopy in the sixth month of the case course.

## Discussion and conclusions

As a rare tumor, bladder paraganglioma accounts for 0.06% of all bladder tumors [3]. The first case in the literature was reported by Zimmerman *et al.* in 1953, and more than 200 cases have been added in the following years [4]. Bladder paraganglioma are derived from the neural crest chromaffin cells that make up the autonomic nervous system, located in the muscle layer or bladder propria. Approximately 10% of bladder paragangliomas are malignant and show local invasion, lymph node spread, or distant metastasis [5].

Bladder paragangliomas are seen in the third decade of life, more frequently in women than in men [6]. Paragangliomas can be functional or nonfunctional by secreting catecholamines. It may cause symptoms such as flushing, hypertension, palpitations, and tremor due to catecholamine release. Macroscopic hematuria can be seen in both functional and nonfunctional paragangliomas.

Bladder paraganglioma should be considered in the differential diagnosis if symptoms associated with catecholamine discharge are found in patients with a solitary lesion in the bladder. However, symptoms related to catecholamine release may not be seen in every case of bladder paraganglioma. By manipulating the tumoral lesion, symptoms may or may not be observed at all. Therefore, cystoscopy should be performed in every patient and diagnostic biopsy should be performed.

Imaging methods do not have superiority in the diagnosis of bladder paraganglioma from other bladder solitary tumors. Metaiodobenzylguanidine scintigraphy has high specificity, but less sensitivity than magnetic resonance imaging in the diagnosis [7]. Ultrasound and contrast-enhanced CT are useful in terms of relationship with neighboring organs and tissues and detection of metastases.

The main goal of treatment is the complete removal of the tumor. There are many surgical options with the use of open, laparoscopic, and endoscopic methods separately and in combination. Hemodynamic instability may develop during tumor manipulation. To prevent this situation, alpha-blocker treatment should be started 3 weeks before the operation.

There is no clinical consensus on postsurgical follow-up. In the literature, tumor recurrence and metastasis have been reported 10 years later. In addition, the criteria for malignancy were not clear on histological evaluation. Therefore, imaging and cystoscopy-based follow-up should be applied.

The rarity of paragangliomas causes the lack of sufficient information on how to treat and follow-up. At the diagnosis stage, clinical findings and imaging methods do not distinguish them from other bladder malignancies. Endoscopic procedures are mostly performed for diagnostic purposes and partial cystectomy is the most common treatment.

Our study aimed to contribute to disease management in the future, with clinical presentation, diagnosis, and treatment strategies.

## Data Availability

The data that support the findings of this study are available from the corresponding author upon reasonable request.

## References

[1] Scoazec JY, Couvelard A. Classification of pancreatic neuroendocrine tumours: changes made in the 2017 WHO classification of tumours of endocrine organs and perspectives for the future. Ann Pathol. 2017;37(6):444–56. 10.1016/j.annpat.2017.10.003 29169836

[2] Zhu X, *et al*. Bladder paraganglioma managed with transurethral holmium laser resection: a case report. Medicine (Baltimore). 2021;100(34): e26909. 10.1097/MD.0000000000026909 PMC8389942 34449457

[3] Johnson JT, *et al.* Micturition syncope secondary to urinary bladder paraganglioma. BMJ Case Rep. 2020; 13(3). 10.1136/bcr-2019-233556 PMC7069265 32169989

[4] Zimmerman IJ, Biron RE, Macmahon HE. Pheochromocytoma of the urinary bladder. N Engl J Med. 1953;249(1):25–6. 10.1056/NEJM195307022490106 13063676

[5] Pelegrín-Mateo FJ, *et al*. Bladder paraganglioma: report of two cases and a literature review. Arch Esp Urol. 2021;74(4):445–9. 33942738

[6] Hermi A, *et al*. Functional bladder paraganglioma treated by partial cystectomy. Case Rep Urol. 2019;2019:4549790. 10.1155/2019/4549790 PMC6925741 31886013

[7] Bhalani SM, Casalino DD, Manvar AM. Paraganglioma of the bladder. J Urol. 2011;186(1):279–80. 10.1016/j.juro.2011.04.032 21600612

